# Reconstruct gene regulatory network using slice pattern model

**DOI:** 10.1186/1471-2164-10-S1-S2

**Published:** 2009-07-07

**Authors:** Yadong Wang, Guohua Wang, Bo Yang, Haijun Tao, Jack Y Yang, Youping Deng, Yunlong Liu

**Affiliations:** 1 School of Computer Science and Technology, Harbin Institute of Technology, Harbin, Heilongjiang, 150001, PR China; 2 Center for Computational Biology and Bioinformatics, Indiana University School of Medicine, Indianapolis, Indiana, 46202, USA; 3 Center for Medical Genomics, India University School of Medicine, Indianapolis, Indiana, 46202, USA; 4 Division of Biostatistics Department of Medicine, Indiana University of School of Medicine, Indianapolis, Indiana, 46202, USA; 5 Harvard Medical School, Harvard University, P.O. Box 400888, Cambridge, Massachusetts, 02115, USA; 6 SpecPro Inc, Vicksburg, Mississippi 39180, USA; 7 Department of Biological Science, University of Southern Mississippi, Hattiesburg, Mississippi, 39406, USA

## Abstract

**Background:**

Gene expression time series array data has become a useful resource for investigating gene functions and the interactions between genes. However, the gene expression arrays are always mixed with noise, and many nonlinear regulatory relationships have been omitted in many linear models. Because of those practical limitations, inference of gene regulatory model from expression data is still far from satisfactory.

**Results:**

In this study, we present a model-based computational approach, Slice Pattern Model (SPM), to identify gene regulatory network from time series gene expression array data. In order to estimate performances of stability and reliability of our model, an artificial gene network is tested by the traditional linear model and SPM. SPM can handle the multiple transcriptional time lags and more accurately reconstruct the gene network. Using SPM, a 17 time-series gene expression data in yeast cell cycle is retrieved to reconstruct the regulatory network. Under the reliability threshold, *θ *= 55%, 18 relationships between genes are identified and transcriptional regulatory network is reconstructed. Results from previous studies demonstrate that most of gene relationships identified by SPM are correct.

**Conclusion:**

With the help of pattern recognition and similarity analysis, the effect of noise has been limited in SPM method. At the same time, genetic algorithm is introduced to optimize parameters of gene network model, which is performed based on a statistic method in our experiments. The results of experiments demonstrate that the gene regulatory model reconstructed using SPM is more stable and reliable than those models coming from traditional linear model.

## Introduction

Gene expression arrays, which measure mRNA expression levels of thousands of genes simultaneously, make it possible to understand the complexities of biological system. By using the gene expression array in a time series paradigm, we can study the effects of certain treatments, diseases, developmental stages and drug responses on gene expression. Moreover, the underlying gene regulatory networks can be reconstructed by collecting and analyzing expression array data. Therefore, identifying gene regulatory networks from gene-expression data is now an extremely active research field.

In previous studies, the time-series data of gene expression arrays are very useful for investigating regulatory interactions between genes. Cho [1] published a 17-point time series data set measuring the expression levels of 6601 genes for yeast saccharomyces cerevisiae, obtained by Affymetrix hybridization array. Using RT-PCR, Wen [2] generated 9-point time series data for the expression levels of every U2 gene involved in the rat nervous system development. But an important and challenging problem is how to discover the associated functions of genes based on this huge amount of data. Many approaches are proposed for gene regulatory networks modeling from gene expression data, such as Boolean network [3-6], linear model [7-9], Bayesian networks [10-14], neural networks [15,16], differential equations [17-19], models including stochastic components on the molecular level [20], and so on. Those models can be classified into fine-grained and coarse grained approaches. The fine-grained approach is based on detailed biochemical knowledge and complex networks of biochemical reactions, whose purpose is to make those models to fit the expression data completely. Linear model is one of the major fine-grained models. However, gene expression array usually contains noises, which lead to breach of feasibility and reliability of fine-grained method. Because small fluctuations in the data may lead to modeling errors of fine-grained approach, it is essential to construct coarse-grained descriptions of gene regulatory networks for studying large scale gene networks. Instead of focusing on the exact biochemical reactions, coarse-grained approaches analyze large gene networks at some intermediate levels by using macroscopic variables in a global pattern. Boolean network model is one of the typical coarse-grained models. However, gene expression levels tend to be continuous rather than discrete, and discretization can lend to a large loss of information.

In this paper, a novel Slice Pattern Model (SPM) is proposed to identify gene regulatory networks from gene expression arrays mixed with noise data. It is a hybrid approach that combines linear model and pattern recognition. In general, models have more variables than available data points. Therefore, a genetic algorithm (GA) is introduced to optimize the parameters of regulation in gene networks [7,21]. We aim at providing a method that can fulfill the experimental requirements against stochastic noise of gene expression data, and identify more interaction information between genes for reconstruction of gene regulatory network. Using SPM, We present a comprehensive identified gene regulatory network from the time-series gene expression arrays of saccharomyces cerevisiae in cell cycle stage. The results demonstrate that our approach is able to identify the time of transcriptional lags between potential regulators and their targets. At the same time, it is robust and stable to reconstruct gene regulatory networks from experimental data mixed with noise.

## Methods

### Rationale

Traditional linear model [9] defined in Equation (1) is based on the fact that gene expression levels tend to be continuous rather than discrete. It assumes that the interaction between genes is linear correlation. Therefore, it is a continuous expression data modeling to find the subset of genes whose weight sum most correlates with the expression levels of a specific gene.

(1)

where *N *is the number of gene in gene network, *x*_*i *_denotes the expression level of gene *i *at time point *t*_*k*+1_, weight *w*_*ji *_indicates the influence of gene *j *regulated by gene *i*, *T *is the number of time point in gene expression data, and Δ*t *represents the average time of interaction response. Given a set of time equidistantly expression data, the weights *w*_*ji *_can be solved by using linear algebra when the number of data points is more than the number of variables.

The task of identifying gene regulatory networks is to optimize parameters, and minimize the residual between the linear model and the gene expression data, which is showed in Equation (2).

(2)

where *y(t*_*k*+1_) is the expression level of gene i at time point *t*_*k*+1 _in gene expression data,  denotes the expression level of gene i at time point *t*_*k*+1 _in linear model.

However, linear model only considers that interaction response takes place between genes with one average time delay. In fact, some interactions between genes possibly take multiple transcription time lags, and the transcription time lags are variable for different regulatory relationships in gene networks [22,23]. Moreover, linear model aims at training gene network models to fit the expression data exactly. In fact, the available expression data is usually mixed with noise, and small fluctuations (noise) on data may induce the random variation of external parameters and chance events in biochemical reactions [24]. The biological noise or measurement variability might change gene expression levels and affects the linear model which determines the value of the weights in gene model. Therefore, the linear model might fail in reconstructing unreal regulatory relationships for fitting the gene expression data with noise, which retards the reliability of reconstruction for gene network.

### Slice pattern model to reconstruct the gene regulatory networks

**Figure 1:**
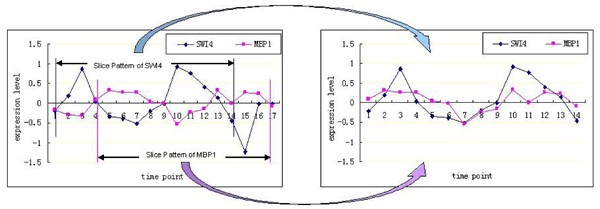
**Strong statistical correlation between the initial expression changes of SWI4 and MBP1 using a 30 min time difference, 3 time (unit) lags**.

In order to solve the limitations of linear model, we propose a new method, slice pattern model (SPM), to reconstruct the gene regulatory networks from gene expression data mixed with noise. SPM is designed to identify a set of genes whose expression levels change not only at the next time point, but also at more time lags. Some regulatory interactions take place with more time lags, for example, the known relationship SWI4 → MBP1 shows significant statistical correlation when transcriptional time lag is identified as three time units (three time units = 30 min) [22] (Figure 1).

For the time-series expression data, the local regulation relationship is considered, and the gene expression data in the multi consecutive times is divided into series slices with *k-*size sliding window. Let a time-series set G(*g*_1_, *g*_2_,..., *g*_*T*_) represents a set of gene expression data in multi data points. When a sliding window with size *k *slides on *G *from point *g*_1 _to *g*_*T*-*k*+1_, it will generate (*T-k *+1) slices for a gene. This operation is performed on each gene expression profile, and a total of *N *× (*T*-*k *+ 1) slices are formed a gene expression dataset with N genes. A matrix of expression slice is constructed according to the matrix of gene expression dataset.

**Figure 2:**
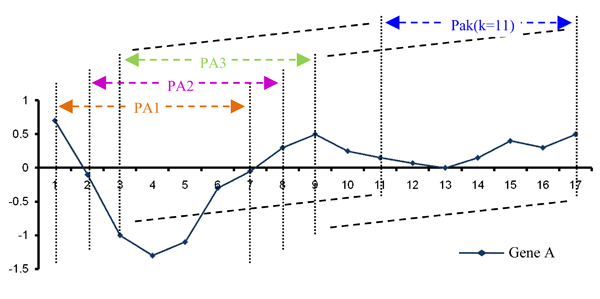
**The series slice pattern (PA1, PA2,..., PA11) in Gene A with 17 data points, and the size k of sliding window is 7**.

For further analysis, the rank patterns of gene expression levels in each slice are extracted, and those slice patterns indicate the feature of a gene. Considering a slice S with k data,

(3)

the ranking pattern can be defined as *P*(*S*) = (*RS*(*s*_1_), *RS*(*s*_2_),..., *RS*(*s*_*k*_)), where *RS*(*s*_*i*_) denotes the rank of *S*_*i *_in *P*(*S*). Thus, each gene can be represented as a set of frameworks combines with a series slice patterns (Figure 2). With the help of pattern recognition on gene expression levels, some small fluctuations (noises) on data have been filtered.

In the current study, we extend the traditional linear model to solve the problem that traditional linear model does not work on multiple time lags. The model named slice pattern model (SPM) use the following formulation:

(4)

where *τ*_*j *_is the time lag of regulatory interaction between gene j and gene i. *x*_*i*_*(t*_*k*_) is the expression level of gene *i *at time *t*_*k*_. *η *is the max time lag with biological meaning, and *L *is the size of gene set which regulate gene *i*.

Since the real expression array data are usually mixed with noises, the comparison between two genes is always disturbed by noise. For ranking pattern in each slice of our method, the spearman rank correlation (SRC) is introduced to estimate the similarity between two patterns, which has been used to assist in measuring the similarity between two genes [25].

The SRC score between two slice pattern *S *and *S' *is given by the following equation:

(5)

where *RS(s*_*i*_) is the rank of *s*_*i *_in the profile (*s*_1_,..., *s*_*k*_). The SRC satisfies -1 ≤ SRC(*S*, *S'*) ≤ 1 for all *S*, *S'*. The SRC score "-1" represents the complete opposite for the two rank patterns. So we can identify the similarity between two patterns according to the SRC score. It is fit for handling distinct fluctuation data mixed in one point, which takes place by accident in a microarray experiment.

Thus, gene regulatory network identifying becomes to an question to optimize a set of parameters *w*_*ji*_, and to maximize the SRC between SPM and the gene expression data.

(6)

where *O*_*i*_(*j*) is the *j-th *slice pattern of gene *i *in gene expression data, and *S*_*i*_(*j*) is the *j-th *slice pattern of gene *i*.

For optimizing parameters of gene network to satisfy those genes slice, an improved genetic algorithm (GA) is introduced to optimize the model that SPM retrieved from gene expression data. The genetic algorithm (GA) was formally introduced in the 1970s by John Holland, which has been used in many research fields as an optimization method [7]. In our case, the parameters of gene regulatory network (including regulatory direction, weight and time lag) are optimized by GA. The iterative procedure is summarized in Additional file 1: The procedure of Slice Pattern Model.

Since the number of gene N is always more than the number of time point T in most publicly available gene expression data set, repeated modeling is needed to get a statistical result. The genetic algorithm is a stochastic algorithm, so the result of each GA run is not same. In current study, if a gene connection is presented more than the threshold value *θ *in repeated modeling, the connection is added into a final gene regulatory network with the value of parameters equal to the average of those in the repeated modeling.

## Results

In this study, we test the performances of linear model and slice pattern model in an artificial gene network. Then, in order to evaluate the feasibility of SPM on real gene expression array data, a yeast cell cycle gene network with nine specific genes is reconstructed by SPM, and verified by comparing with established relationships in previous investigations.

### The performance of SPM method

**Figure 3:**
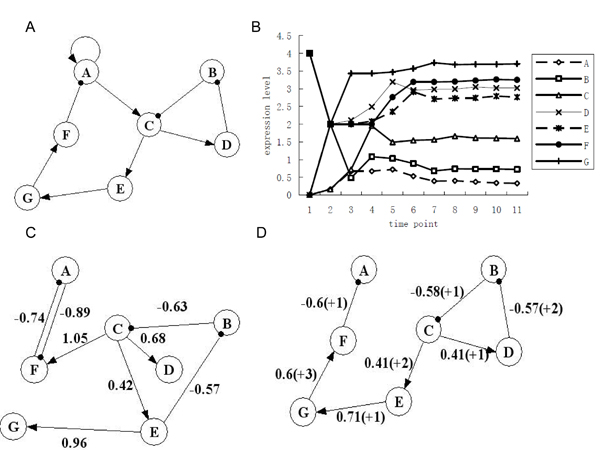
**Simulations of an artificial gene network in different models**. (A) Original artificial gene regulatory network, arrow line denotes the stimulation, dot line denotes the inhibition; (B) Time series expression profiles of each gene in artificial gene network; (C) Reconstructed gene regulatory network using Linear Model; (D) Reconstructed gene regulatory network using Slice Pattern Model.

We take an artificial gene network with known structure (Figure 3A) coming from Ando and Iba's experiments [7] to test the performances of linear model and SPM. Each method is taken to run 10 times independently for modeling gene network, the threshold *  θ *is set as 60%.

Firstly, initial condition and status (Table 1) are set for the gene network to produce a time series gene expression data. Two gene regulatory networks with seven genes are reconstructed by linear method and SPM independently from the time series gene expression data (Figure 3B, C, D). In the result shown in Figure 3, those regulatory relationships with one transcriptional time lag (such as B-C, C-D etc.) can be identified exactly by linear model. The traditional linear model does not work when interaction responses between genes take variable multiple transcription time lags for different regulatory relationship in gene network. Moreover, the aim of linear model is to train gene network models to fit the expression data exactly. Therefore, the linear model might fail in reconstructing unreal regulatory relationships for fitting the gene expression data, for example, unreal regulatory relationships A-F, C-F and E-B, which retard the reliability of reconstruction for gene network. Comparing with traditional linear model, slice pattern model (SPM) can handle the multiple transcriptional time lags. SPM identifies the time lags while it reconstructes the gene network.

**Table 1:** Benchmark result of the cascade oscillators model

Gene pair	Regulatory weight	Time (unit) lag(s)	Initial expression level of regulator
A-A	0.5	3	0.0
A-C	0.5	1	0.0
B-C	-0.8	1	4.0
C-D	0.7	1	0.0
C-E	0.5	2	0.0
D-B	-0.5	2	4.0
E-G	0.9	1	0.0
F-A	-0.8	1	4.0
G-F	0.4	3	0.0

### Identification of gene regulatory network in yeast cell cycle

A gene expression dataset, yeast cell cycle time-series gene expression arrays which is obtained from Cho [1], is taken to evaluate the feasibility of SPM. The data set contains 17 time points with relatively small time intervals (10 min), thus the data is ideal for testing the approach. In our case, for studying the reliability of SPM, we focus on nine specific factors, MBP1, SWI4, SWI6, MCM1, FKH1, FKH2, NDD1, SWI5, and ACE2, which control the transcription of cell cycle genes. Many previous studies [26-28] using different approaches have established some regulatory relationships for these nine transcription factors (TFs).

**Figure 4:**
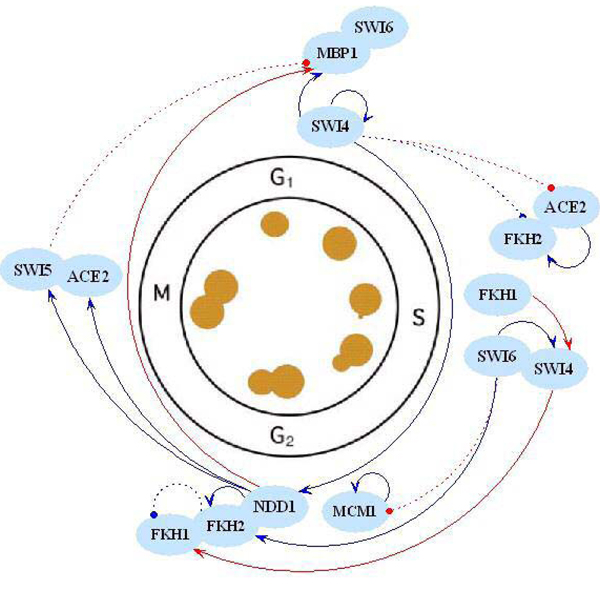
**Reconstructed transcriptional regulatory network of the yeast cell cycle**. The stimulating interactions between the transcription factors and their target genes are indicated by arrow lines, and inhibiting interactions are indicated by dashed lines. Blue lines represent known regulatory relationships that identified by previous studies and red lines represent potential regulatory relationships that need further examination to be identified.

In this study, the modeling process had been run 20 times independently to reconstruct the gene network. The result is shown in table 2, in which time lags of gene pair are the sum of time lags in multi runs. The frequency of each gene pair's regulatory relationship in 20 repeat modeling can be estimated using "Time lags" divided by "Repeats", which could be the reliability indicator of gene regulatory relationship. The average time lag and regulatory effect of each pair is summarized in Table 3. A simple gene network of yeast cell cycle with 9 TFs is reconstructed (Figure 4), in which the regulatory relationships are identified by filtering those connections whose reliability is below *θ *= 55%.

**Table 2:** Result of modeling a simple yeast cell cycle gene network with SPM

		SWI4	NDD1	ACE2	SWI5	MCM1	SWI6	FKH2	MBP1	FKH1
SWI4	Time lags	19	57	24	18	20	14	23	39	24
	Repeats	12	19	11	10	9	8	13	13	12
NDD1	Time lags	19	21	47	33	20	15	18	21	11
	Repeats	9	7	16	13	8	6	12	12	4
ACE2	Time lags	6	22	23	25	11	28	26	15	9
	Repeats	4	10	7	7	5	8	11	5	3
SWI5	Time lags	16	4	24	23	21	9	13	36	7
	Repeats	4	4	8	7	10	3	5	14	3
MCM1	Time lags	13	17	11	13	21	24	14	20	10
	Repeats	10	9	4	6	14	10	5	10	5
SWI6	Time lags	41	10	22	12	25	6	40	14	11
	Repeats	11	4	10	6	12	2	14	7	7
FKH2	Time lags	11	11	21	29	17	10	8	9	50
	Repeats	7	5	8	9	6	4	3	7	18
MBP1	Time lags	9	14	2	8	6	13	15	15	5
	Repeats	3	4	1	5	2	5	5	7	2
FKH1	Time lags	15	8	6	13	13	8	3	6	3
	Repeats	11	3	3	7	6	4	2	2	2

**Table 3:** The regulatory relationships between genes in yeast cell cycle network

Regulator	Target	Activation/Inhibition	Average time-lag	Reliability
SWI4	NDD1	A	3	95%
FKH2	FKH1	I	2.8	90%
NDD1	ACE2	A	2.9	80%
SWI5	MBP1	I	2.6	70%
MCM1	MCM1	A	1.5	70%
SWI6	FKH2	A	2.9	70%
SWI4	FKH2	I	1.8	65%
SWI4	MBP1	A	3	65%
NDD1	SWI5	A	2.5	65%
SWI4	SWI4	A	1.6	60%
SWI4	FKH1	A	2	60%
NDD1	FKH2	A	1.5	60%
NDD1	MBP1	A	1.75	60%
SWI6	MCM1	I	2.1	60%
SWI4	ACE2	I	2.2	55%
ACE2	FKH2	A	2.4	55%
SWI6	SWI4	A	3.7	55%
FKH1	SWI4	I	1.4	55%
SWI4	SWI5	I	1.8	50%
ACE2	NDD1	I	2.2	50%
SWI5	MCM1	I	2.1	50%
MCM1	SWI4	A	1.3	50%
MCM1	SWI6	A	2.4	50%
MCM1	MBP1	A	2	50%
SWI6	ACE2	I	2.2	50%
.	.	.	.	.
.	.	.	.	.
.	.	.	.	.

Previous studies [26-36] identified the transcriptional regulators for most cyclin genes. SBF (SWI4/SWI6) and MBF (MBP1/SWI6), which are active during late G1, both regulate NDD1. NDD1 does not directly bind to DNA but interacts with FKH1 or FKH2, both of which bind directly to DNA, and NDD1 is a limiting component of the complex that activates G2/M genes. MCM1 and FKH2 are bound to promoters of G2/M genes throughout the cell cycle, and activation of G2/M genes depends on recruitment of NDD1. The MCM1/FKH2/NDD1 complex regulates SWI5 and ACE2. SWI5, ACE2, and MCM1 activate M/G1 genes. MCM1 binds to the SWI4 promoter and contributes to its activation in M/G1, leading to accumulation of the SWI4 in G1. SWI4 transcription is further regulated in late G1 by both SBF and MBF. Thus, the serial regulation of cell cycle regulators occurs throughout the cycle, forming a fully connected regulatory network.

Our results confirm these observations and further identify the details of regulation relationships, such as the active/inhibitive interaction with transcriptional lags. Some novel interactions reconstructed by SPM are needed to be studied further. ACE2 and SWI5 are transcription factors that function at the M/G1 boundary [28,37]. However, we find there is a tight correlation between SWI4 and ACE2, and a similar situation exists for FKH2 (Figure 4). Summary of previous evidence for regulation of cell cycle transcription shows that transcriptional control in S and S/G2 stage is less well characterized, but some studies suggest the involvement of SBF and FKH1/FKH2 [26,28,38]. Our finding indicates that the latter might be more reasonable.

## Discussion

Linear model gives a description of the continuous expression data modeling, which reflect the property of gene expression levels tending to be continuous. Reconstruction of gene regulatory network is a reverse engineering to infer all of the unknown parameters in linear model from gene expression data. However, due to the limitations of experiment, such as the multiple transcriptional time lags and lack of data points, the traditional linear models lead to misleading modeling. We showed the unreliability of linear model when inferring gene network with variable multiple transcriptional time lags. In fact, many studies have demonstrated that some interactions between genes take more than one unit of time lag, and the transcriptional lag is diversity.

In our approach, we suggest that the time lag is determined, and those time lags far from biologically meaning will be removed during modeling (e.g. those time lags that not exceeding 5 are regarded as being biologically meaning). And feature retrieved from expression data may reduce noise interference to a certain extent.

For identifying gene regulatory networks, the parameters of gene networks are optimized via genetic algorithm (GA). The novel development of genetic operations is implemented different from other methods. Our approach reconstructs a model that has the optimal pattern matching to the expected slice patterns.

Along with the analysis of experiments discussed above, we suggest that the pattern matching to modeling of gene network may enhance the performance. According to the result of experiment on yeast cell cycle time-series gene expression data, three features of the resulting network model are notable. First, the stability of the gene regulatory model reconstructed using SPM is better than those models coming from traditional linear model. Second, SPM can determine not only the influence of regulator on target gene, but also the time lags of regulation. Finally, and most importantly,  the reconstruction of the gene regulatory networks is automatic and required no prior knowledge of the direction of regulation. SPM represents a general method for constructing the regulatory networks from the time series expression data.

## Conclusion

We present a model-based computational approach, Slice Pattern Model (SPM), to identify gene regulatory networks from time series gene expression arrays. By testing the performance in an artificial gene network, SPM can handle the multiple transcriptional time lags and more accurately reconstruct the gene networks than traditional linear model. A 17 time-series gene expression data in yeast cell cycle is used to reconstruct the regulatory network. The results demonstrate that the gene regulatory model reconstructed by SPM is more stable and reliable than those models coming from traditional linear model.

## Competing interests

The authors declare that they have no competing interests.

## Authors' contributions

YW, GW, YB and YL contributed to the design of the study. GW, YB and YL designed and performed the computational modelling and drafted the manuscript. YW, HT, YYJ, YD and YL participated in coordination, discussions related to result interpretation and revision of the manuscript. All the authors read and approved the final manuscript.

## Supplementary Material

Additional file 1 The procedure of slice pattern model. Click here for file

## References

[B1] Cho RJ, Campbell MJ, Winzeler EA, Steinmetz L, Conway A, Wodicka L, Wolfsberg TG, Gabrielian AE, Landsman D, Lockhart DJ (1998). A genome-wide transcriptional analysis of the mitotic cell cycle. Mol Cell.

[B2] Wen X, Fuhrman S, Michaels GS, Carr DB, Smith S, Barker JL, Somogyi R (1998). Large-scale temporal gene expression mapping of central nervous system development. Proc Natl Acad Sci USA.

[B3] Sahoo D, Dill DL, Gentles AJ, Tibshirani R, Plevritis SK (2008). Boolean implication networks derived from large scale, whole genome microarray datasets. Genome Biol.

[B4] Akutsu T, Miyano S, Kuhara S (1999). Identification of genetic networks from a small number of gene expression patterns under the Boolean network model. Pac Symp Biocomput.

[B5] Liang S, Fuhrman S, Somogyi R (1998). Reveal, a general reverse engineering algorithm for inference of genetic network architectures. Pac Symp Biocomput.

[B6] Linden R, Bhaya A (2002). Reverse engineering of genetic networks using variable length genetic algorithms with a Boolean network model. Intelligent Engineering Systems Through Artificial Neural Networks.

[B7] Ando S, Iba H (2001). Inference of Gene Regulatory Model by Genetic Algorithms. Proc IEEE Congress on Evolutionary Computation: 2001.

[B8] D'Haeseleer P, Wen X, Fuhrman S, Somogyi R (1999). Linear modeling of mRNA expression levels during CNS development and injury. Pac Symp Biocomput.

[B9] van Someren EP, Wessels LF, Reinders MJ (2000). Linear modeling of genetic networks from experimental data. Proc Int Conf Intell Syst Mol Biol.

[B10] Djebbari A, Quackenbush J (2008). Seeded Bayesian Networks: constructing genetic networks from microarray data. BMC Syst Biol.

[B11] Friedman N, Linial M, Nachman I, Pe'er D (2000). Using Bayesian networks to analyze expression data. Journal of Computational Biology.

[B12] Hartemink AJ, Gifford DK, Jaakkola TS, Young RA (2001). Using graphical models and genomic expression data to statistically validate models of genetic regulatory networks. Pac Symp Biocomput.

[B13] Perrin BE, Ralaivola L, Mazurie A, Bottani S, Mallet J, d'Alche-Buc F (2003). Gene networks inference using dynamic Bayesian networks. Bioinformatics.

[B14] Armananzas R, Inza I, Larranaga P (2008). Detecting reliable gene interactions by a hierarchy of Bayesian network classifiers. Comput Methods Programs Biomed.

[B15] Vohradsky J (2001). Neural model of the genetic network. J Biol Chem.

[B16] Weaver DC, Workman CT, Stormo GD (1999). Modeling regulatory networks with weight matrices. Pac Symp Biocomput.

[B17] Brown PO, Botstein D (1999). Exploring the new world of the genome with DNA microarrays. Nat Genet.

[B18] Chen T, He HL, Church GM (1999). Modeling gene expression with differential equations. Pac Symp Biocomput.

[B19] Mestl T, Plahte E, Omholt SW (1995). A mathematical framework for describing and analysing gene regulatory networks. J Theor Biol.

[B20] McAdams HH, Arkin A (1997). Stochastic mechanisms in gene expression. Proc Natl Acad Sci USA.

[B21] Whitehead DJ, Skusa A, Kennedy PJ (2004). Evaluating an Evolutionary Approach for Reconstructing Gene Regulatory Networks. Proceedings of the Artificial Life Conference.

[B22] Zou M, Conzen SD (2005). A new dynamic Bayesian network (DBN) approach for identifying gene regulatory networks from time course microarray data. Bioinformatics.

[B23] Ji L, Tan KL (2005). Identifying time-lagged gene clusters using gene expression data. Bioinformatics.

[B24] Tian T, Burrage K (2003). Stochastic neural network models for gene regulatory networks. The 2003 Congress on Evolutionary Computation.

[B25] Balasubramaniyan R, Hullermeier E, Weskamp N, Kamper J (2005). Clustering of gene expression data using a local shape-based similarity measure. Bioinformatics.

[B26] Lee TI, Rinaldi NJ, Robert F, Odom DT, Bar-Joseph Z, Gerber GK, Hannett NM, Harbison CT, Thompson CM, Simon I (2002). Transcriptional regulatory networks in Saccharomyces cerevisiae. Science.

[B27] Kato M, Hata N, Banerjee N, Futcher B, Zhang MQ (2004). Identifying combinatorial regulation of transcription factors and binding motifs. Genome Biol.

[B28] Simon I, Barnett J, Hannett N, Harbison CT, Rinaldi NJ, Volkert TL, Wyrick JJ, Zeitlinger J, Gifford DK, Jaakkola TS (2001). Serial regulation of transcriptional regulators in the yeast cell cycle. Cell.

[B29] Althoefer H, Schleiffer A, Wassmann K, Nordheim A, Ammerer G (1995). Mcm1 is required to coordinate G2-specific transcription in Saccharomyces cerevisiae. Mol Cell Biol.

[B30] Foster R, Mikesell GE, Breeden L (1993). Multiple SWI6-dependent cis-acting elements control SWI4 transcription through the cell cycle. Mol Cell Biol.

[B31] Koranda M, Schleiffer A, Endler L, Ammerer G (2000). Forkhead-like transcription factors recruit Ndd1 to the chromatin of G2/M-specific promoters. Nature.

[B32] Kumar R, Reynolds DM, Shevchenko A, Goldstone SD, Dalton S (2000). Forkhead transcription factors, Fkh1p and Fkh2p, collaborate with Mcm1p to control transcription required for M-phase. Curr Biol.

[B33] Loy CJ, Lydall D, Surana U (1999). NDD1, a high-dosage suppressor of cdc28-1N, is essential for expression of a subset of late-S-phase-specific genes in Saccharomyces cerevisiae. Mol Cell Biol.

[B34] MacKay VL, Mai B, Waters L, Breeden LL (2001). Early cell cycle box-mediated transcription of CLN3 and SWI4 contributes to the proper timing of the G(1)-to-S transition in budding yeast. Mol Cell Biol.

[B35] Pic A, Lim FL, Ross SJ, Veal EA, Johnson AL, Sultan MR, West AG, Johnston LH, Sharrocks AD, Morgan BA (2000). The forkhead protein Fkh2 is a component of the yeast cell cycle transcription factor SFF. EMBO J.

[B36] Zhu G, Spellman PT, Volpe T, Brown PO, Botstein D, Davis TN, Futcher B (2000). Two yeast forkhead genes regulate the cell cycle and pseudohyphal growth. Nature.

[B37] Doolin MT, Johnson AL, Johnston LH, Butler G (2001). Overlapping and distinct roles of the duplicated yeast transcription factors Ace2p and Swi5p. Mol Microbiol.

[B38] Liu XS, Brutlag DL, Liu JS (2002). An algorithm for finding protein-DNA binding sites with applications to chromatin-immunoprecipitation microarray experiments. Nat Biotechnol.

