# Supplementary material for: Reconstruct gene regulatory network using slice pattern model
Source: BMC Genomics. 2009 Jul 7;10(Suppl 1):S2. doi: 10.1186/1471-2164-10-S1-S2 (PMC2709263; doi:10.1186/1471-2164-10-S1-S2)
Supplement: Additional file 1 — The procedure of slice pattern model. [file 1471-2164-10-S1-S2-S1.pdf]

Slice Pattern Model Based on Genetic Algorithm

Step 1. Extract slices: generate a series slices from time-series gene expression data using a sliding window with k-size.

Step 2. Determining patterns: rank the data in each slice to generate the slice pattern.

Step 3. Represent the genes as a series slice patterns.

Step 4. GA initialization. Initialize the parameters of GA, including the size of colony *C*, the maximal iteration *I*, the expected precision *ε*, mutation rate on weight M_{PS} , mutation rate on regulation direction M_{PS} , mutation rate on transcription time lag M_{PT} and crossover rate on GA chromosome C_P .

Step 5. Randomly produce an initial colony with series chromosomes *D(i)* , *i =1, 2,...,C.*

Step 6. Compute the maximum of fitness

$$F_{MAX} = N \times (T - k + 1)$$

Step 7. Estimate the fitness *F(i)* of individuals in the colony using following equation.

$$\underset{w \in W, \lambda \subset G}{Max} \left\{ \sum_{i=1}^{N} \sum_{j=1}^{T-k+1} SRC(O_i(j), S_i(j)) \right\}$$

Step 8. Produce new generation using selection operation.

if $\frac{F(i)}{F(BestOne)} < Rand(0,1)$ *then*

$D(i) \Leftarrow D(BestOne);$

End if

Step 9. Mutate the chromosomes with M_{PW} , M_{PS} and M_{PT}.

Step 10. Some chromosomes are selected to be crossover.

for i=1 to C

if Rand(0,1)<C_P *then*

m:=Rand[1,C];

```
        n:=Rand[1,C];
        Crossover (D(m),D(n) );
    end if
end for
```

Step 11. Check the Termination-conditions:

```
if I=0 or (FMAX-F(BestOne))<ε
    Return model;
else
    I:=I-1;
    go to step 7.
end if
```
